# Bilateral Carotid-Cavernous Fistulas: An Uncommon Cause of Pituitary Enlargement and Hypopituitarism

**DOI:** 10.1155/2016/6364203

**Published:** 2016-08-29

**Authors:** Anthony Liberatore, Ronald M. Lechan

**Affiliations:** Department of Medicine, Division of Endocrinology, Diabetes and Metabolism, Tupper Research Institute, Tufts Medical Center, Boston, MA 02111, USA

## Abstract

Carotid-cavernous fistulas (CCFs) are rare, pathologic communications of the carotid artery and the venous plexus of the cavernous sinus. They can develop spontaneously in certain at risk individuals or following traumatic head injury. Typical clinical manifestations include headache, proptosis, orbital pain, and diplopia. We report a case of bilateral carotid-cavernous fistulas associated with these symptoms and also with pituitary enlargement and hypopituitarism, which improved following surgical intervention. Arterialization of the cavernous sinus and elevated portal pressure may interfere with normal venous drainage and the conveyance of inhibiting and releasing hormones from the hypothalamus, resulting in pituitary enlargement and hypopituitarism. This condition should be considered in the differential diagnosis of hypopituitarism associated with anterior pituitary enlargement.

## 1. Introduction

Diffuse enlargement of the anterior pituitary gland can be associated with a number of physiological and pathologic clinical conditions. Physiologic enlargement is seen during adolescence and pregnancy and after menopause [1, 2] and can occur with long-standing primary hypothyroidism, hypogonadism, or hypoadrenalism due to thyrotroph, gonadotroph, or corticotroph hyperplasia, respectively [1, 3]. A variety of pathologic conditions also result in diffuse enlargement of the anterior pituitary and can be associated with varying degrees of hypopituitarism including infiltrative and inflammatory disorders such as hemochromatosis, amyloidosis, sarcoidosis, Langerhans cell histiocytosis, lymphocytic and granulomatous hypophysitis, Wegener's granulomatosis, necrotizing infundibular hypophysitis, and Erdheim-Chester disease; infectious disorders including bacterial, fungal, and parasitic disease; and neoplastic disorders including germinoma, metastatic carcinoma, myeloma, lymphoma, and leukemia [4–7]. Other rare causes include ectopic or excess secretion of GHRH or CRH from a ganglioneuroma or carcinoid tumor [8–10] and germline mutations of* PROP1* [11]. Transient pituitary enlargement has also been reported after bilateral cavernous sinus thrombosis [12].

An underrecognized and uncommon cause of pituitary enlargement is vascular congestion from carotid-cavernous fistulas (CCFs). CCFs are rare arteriovenous shunts that permit blood flow from the carotid artery into the cavernous sinus. The cavernous sinus contains a venous plexus that drains the confluent veins emanating from anterior and posterior pituitary. Thereby, arterialized blood in the cavernous sinus can disrupt normal venous pituitary drainage and cause pituitary enlargement [13], hypoperfusion [14], and hypopituitarism [15, 16]. To increase awareness of this disorder and the characteristics of its presentation, we report a case of 53-year-old woman found to have bilateral CCFs while being evaluated for pituitary enlargement and hypopituitarism. The fistulas were repaired surgically resulting in reduction in pituitary size and eventual recovery of her hypothalamic-pituitary-adrenal axis. However she has ongoing central hypothyroidism.

## 2. Case Report

A 53-year-old woman was referred to the emergency department with a left, cranial nerve VI palsy and enlarged pituitary on head MRI. For the three months prior to admission, she had complaints of headaches, sinus pressure, and bilateral eye redness and had been evaluated by several physicians. On these occasions, she was treated for seasonal allergies with short courses of antibiotics and corticosteroids, but without significant improvement in her symptoms. Two weeks prior to admission, while driving, she developed acute-onset diplopia with lightheadedness and vertigo. Her visual symptoms improved if she covered her left eye. These new symptoms lead to referral to an otolaryngologist and then an ophthalmologist who obtained MRI which revealed pituitary enlargement. She was sent to the emergency room following the discovery of the pituitary abnormality.

**Figure 1:**
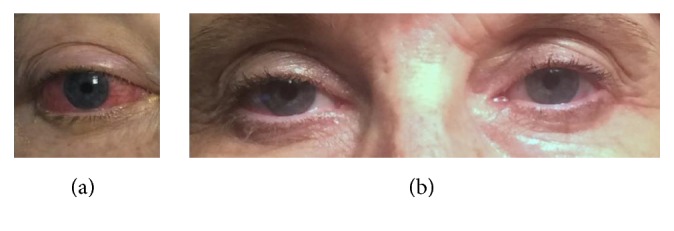
Patient's eye findings on presentation (a) and 1 month after embolization (b). Initial periorbital edema, chemosis, proptosis, and conjunctival injection dramatically improved with coiling.

In the emergency department, she had a notable, isolated, left, cranial nerve VI palsy, periorbital edema, bilateral conjunctival injection with chemosis (Figure 1(a)), and a delayed relaxation phase of her biceps reflex. Given the pituitary enlargement, she was admitted to the Neurosurgery service. Ophthalmology was consulted and their evaluation noted elevated intraocular pressure, 26 mmHg on the right and 21 mmHg on the left (normal 10–20 mmHg). Fundoscopic examination revealed sharp optic discs. Her initial blood pressure was 137/67 mmHg with pulse 65 beats per minute. There was no postural change in her vital signs. Laboratory testing revealed a serum sodium of 142 mmol/L (135–145), potassium 4 mmol/L (3.6–5.1), random cortisol undetectable, and ACTH 5.5 pmol/L. A 250-*μ*g cosyntropin stimulation test was performed the following morning. Basal cortisol was 27.6 nmol/L, 30 minute cortisol 405 nmol/L, and 60-minute maximum 488 nmol/L. TSH was normal at 2.21 mIU/L (0.4–4.8) and free thyroxine was borderline low, 9.78 pmol/L (9–19). Prolactin was elevated at 1.7 nmol/L (0.17–1.3). Other anterior pituitary function tests were normal and age-appropriate (IGF-1 17.03 nmol/L [11–30.5]; FSH 61.4 IU/L [26.7–133.4]). Glucocorticoids and levothyroxine were started.

**Figure 2:**
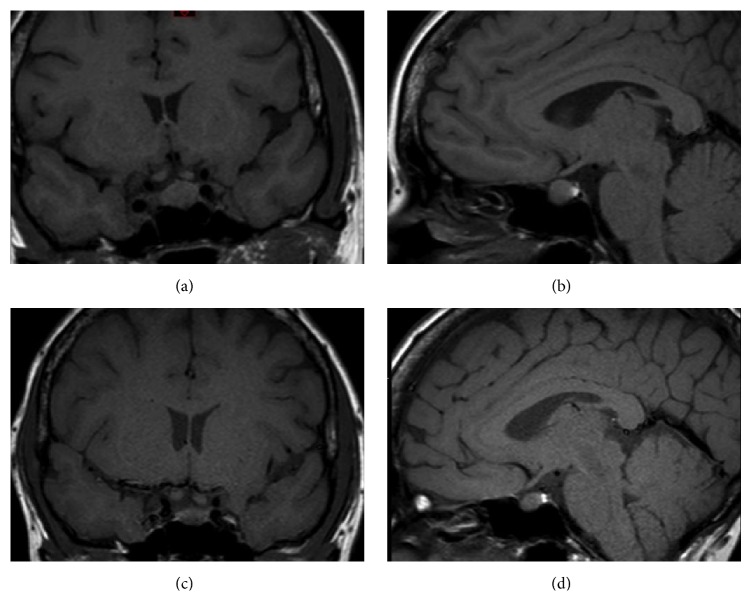
Sella MRI images showing pituitary enlargement at presentation (a, b) and 4 months after embolization (c, d), showing significant reduction in size.

**Figure 3:**
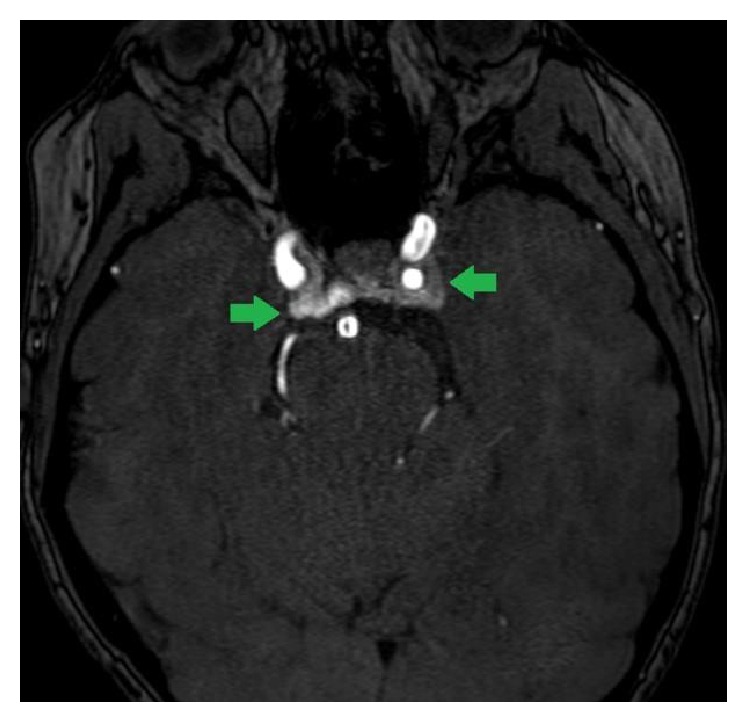
Axial head MRA image demonstrating flow-related contrast from the internal carotid arteries entering the cavernous sinuses (arrows), suggesting bilateral carotid-cavernous fistulas.

Noncontrast head MRI revealed an enlarged pituitary measuring 1.1 cm AP × 0.9 cm superior-inferior × 1.4 cm transverse with upward convexity into the suprasellar cistern (Figures 2(a) and 2(b)). There was no cavernous sinus invasion. Note, however, was made of bilateral, low-signal foci in the cavernous sinuses raising the question of aneurysms or venous flow. MRA confirmed intense early postcontrast enhancement within the cavernous sinuses, adjacent to the internal carotid arteries, consistent with bilateral CCFs (Figure 3). She was taken to the operating room for bilateral endovascular fistula coiling. Within 48 hours, her diplopia improved, steroids were tapered, and she was discharged on hydrocortisone 10 mg with breakfast and 5 mg with supper and a weight-based dose of levothyroxine, 75 mcg once a day.

The patient thereafter was evaluated regularly in the outpatient endocrine clinic at follow-up appointments. At 1 month, significant improvements in eye signs were noted (Figure 1(b)). A head MRI at 4 months showed that the pituitary had decreased in size to 1.3 cm AP × 0.7 cm superior-inferior × 1.2 cm transverse (Figures 2(c) and 2(d)). An early morning cortisol checked 24 hours after the last dose of hydrocortisone was 444 nmol/L and FT4 was 19.3 pmol/dL. Prolactin had decreased to 0.65 nmol/L. As it appeared her adrenal and thyroid axes were recovering; hydrocortisone and levothyroxine were discontinued. Two weeks later, thyroid function tests off of levothyroxine showed persistent central hypothyroidism (TSH 0.87 mIU/L, FT4 7.7 pmol/L, and TT3 1.25 pmol/L [1.08–3.08]) and therefore, levothyroxine was resumed. A second 250 *μ*g cosyntropin stimulation test was performed to further evaluate the hypothalamic-pituitary-adrenal axis. Baseline cortisol was 182 nmol/L, 30-minute cortisol was 477 nmol/L, and 60-minute cortisol was 571 nmol/L. An overnight metyrapone test was then performed to confirm recovery of the pituitary-adrenal axis. After a late night dose of metyrapone, 35 mg/kg, the patient returned for early morning blood work. Morning ACTH was 62 pmol/L with 11-deoxycortisol level >300 nmol/L, demonstrating normal adrenal function and recovery of the pituitary-adrenal axis. While the shunt was cured by embolization, the requirement for levothyroxine suggested residual anterior pituitary dysfunction.

Although the patient was noted to have joint laxity and skin features suggestive of Ehlers-Danlos syndrome, genetic testing for this condition was negative.

## 3. Discussion

The cavernous sinuses contain the internal carotid artery, cranial nerves III, IV, V1, V2, and VI, sympathetic fibers, and a venous plexus that drains the confluent veins emanating from anterior and posterior pituitary [13]. Carotid-cavernous fistulas are rare arteriovenous shunts that permit blood flow from the carotid artery into the cavernous sinus. Abnormal flow into the cavernous sinus due to a carotid-cavernous fistula can disrupt normal venous pituitary drainage and can be acute or subacute, depending on the rate of blood flow.

CCFs can be classified into types A, B, C, or D by angiographic criteria [17]. Type A is a direct shunt between the internal carotid artery and the cavernous sinus [17]. Type B is a dural shunt between the meningeal branches of the internal carotid artery and the cavernous sinus [17]. Type C is a dural shunt between meningeal branches of the external carotid artery and the cavernous sinus [17]. Type D is a simultaneous shunt between the meningeal branches of both the internal and external carotid arteries and the cavernous sinus [17]. Type A CCFs account for approximately 75–80% of all CCFs [18]. They are high-flow shunts that typically result from basilar skull fracture, although they can occur from the spontaneous or iatrogenic rupture of a carotid aneurysm within the cavernous sinus [17–20]. Signs and symptoms of a high-flow, type A CCF include acute-onset headache, diplopia, proptosis, chemosis, orbital pain, and orbital bruit [18]. Types B, C, and D are low-flow shunts and account for approximately 25% of all CCFs. These typically have symptoms similar to direct CCFs but with a more insidious onset [18]. Patients are often treated for myriad conditions from keratoconjunctivitis to presumed thyroid ophthalmopathy to idiopathic orbital inflammatory disease before the dural shunt is diagnosed. Visual loss is more common with high-flow shunts but can occur in 20–30% of low-flow shunts due to ischemic optic neuropathy, chorioretinal dysfunction, or uncontrolled glaucoma, due to elevated venous pressure and, in turn, intraopthalmic pressure [21]. Predisposing factors in the development of spontaneous CCFs include connective tissue disorders such as Ehlers-Danlos type IV, fibromuscular dysplasia, and pseudoxanthoma elasticum, hypertension, atherosclerosis, and pregnancy [18, 21]. Between 10 and 60% of types B–D shunts can resolve spontaneously, possibly due to thrombosis of the affected area of the cavernous sinus [17]. Noninvasive imaging by CT, MR, or CT/MR angiography is often done as a first-line study in patients with symptoms suggestive of CCF [18]. Findings include cavernous sinus enlargement, proptosis, extraocular muscle enlargement, or superior ophthalmic vein dilation [18]. The gold-standard diagnostic test is catheter cerebral angiography, which carries a morbidity rate of less than 1% when done by experienced neuroradiologists [21]. First-line treatment of CCF is transarterial or transvenous coil or liquid embolization to occlude flow through the fistula while maintaining normal flow in the carotid artery [18]. The possibility of adverse events, however, is significantly higher in patients with underlying connective tissue disorders [21]. In one series of Ehlers-Danlos patients, for example, mortality following treatment of CCFs was as high as 59%, 23% of which was directly due to diagnostic or therapeutic procedures themselves [21, 22]. These patients are at increased risk due friability of vessels, propensity to develop arterial dissections, aneurysms, and rupture due to type III collagen defects in the vessel walls [22, 23].

The largest treatment series of indirect, dural CCF cases described 135 patients, followed for an average of 56 months [24]. Patients were mostly female, 73%, with a mean age of 60 years [24]. Presenting signs and symptoms, in order of declining frequency, were arterialization of conjunctival veins (93%), chemosis (87%), proptosis (81%), diplopia with ophthalmoparesis (68%), cranial bruit (49%), retroorbital headache (34%), elevated intraocular pressure (34%), and diminished visual acuity (31%) [24]. In the patients who underwent endovascular vascular surgery, there was a cure rate of 90% [24].

There are rare case reports in the literature of hypopituitarism caused by carotid artery aneurysms. A recent review by Hanak et. al. [25] identified 40 cases of cerebral aneurysms with intrasellar extension, 90% of which were of the ICA. In this series, endocrinopathy was noted in 57% of unruptured aneurysms, 40% of aneurysms that later ruptured, and 83% of aneurysms associated with concurrent pituitary adenomas. Elevated prolactin was the most common finding (90%), followed by gonadotropin deficiency (82%), ACTH deficiency (70%), and TSH deficiency (60%) [25]. Most cases appear to be due to mass effect directly on the pituitary, stalk, or hypothalamus [25–28]. A series of 4087 patients diagnosed with hypopituitarism estimated that the etiology was from an intrasellar aneurysm in 7 patients or 0.17% [26]. The two prior English-literature case reports of hypopituitarism associated with CCFs also report that direct mass effect from the fistulas on either the stalk [15] or pituitary gland itself [16] was a likely etiology for pituitary dysfunction.

Anterior pituitary dysfunction in this patient, however, would not appear to be due to a space occupying mass as seen with aneurysms. More likely, it was due to impaired venous drainage from the pituitary and/or hypothalamus due to increased venous pressure in the confluent veins and/or portal veins, the latter interfering with conveyance of hypothalamic releasing/inhibitory factors to the anterior pituitary. Venous congestion was also likely responsible for the enlargement of the anterior pituitary. Normalization of cortisol and prolactin following correction of the AV shunt in our patient supports this hypothesis, although her ongoing central hypothyroidism raises concern that this condition can result in permanent deficits. The paucity of cases reporting hypopituitarism [15, 16] in association with CCF suggests that it is either uncommon or an underrecognized complication.

Anterior pituitary enlargement and palsy of cranial nerve VI in this patient initially raised the possibility of an inflammatory disorder of the pituitary or a pituitary adenoma. Indeed, hypophysitis, particularly granulomatous hypophysitis, can present as a sellar mass associated with cranial nerve palsies due to involvement of the adjacent cavernous sinus [29] as can occur with other inflammatory disorders of the pituitary including Sjögren's syndrome [30, 31], sarcoidosis [32], Wegener's granulomatosis [33, 34], tuberculosis [35], syphilis [36, 37], and fungal infections [38]. Langerhans histiocytosis, germinoma, and lymphoma may also be associated with similar findings but often associated with central diabetes insipidus [39–41]. A distinguishing feature that separates these disorders from individuals with CCF, however, is the presence of proptosis, conjunctival chemosis, and/or corkscrew episcleral blood vessels which occurs in the majority of cases as was present in the case presented herein [18].

In conclusion, patients suspected of having a CCF should have an assessment of anterior pituitary function, particularly of their thyroid and adrenal axes. If deficiencies are detected, these patients should be treated with hormone replacement therapy to minimize the possibility of complications. After a successful intervention, pituitary function should be reassessed because at least some recovery is possible over the ensuing months. Carotid-cavernous fistula should be considered in the differential diagnosis of pituitary enlargement on imaging studies, particularly when associated with orbital congestion.
